# Development, Characterization Optimization, and Assessment of Curcumin-Loaded Bioactive Self-Nanoemulsifying Formulations and Their Inhibitory Effects on Human Breast Cancer MCF-7 Cells

**DOI:** 10.3390/pharmaceutics12111107

**Published:** 2020-11-18

**Authors:** Mohsin Kazi, Fahd A. Nasr, Omar Noman, Abdulrahman Alharbi, Mohammed S. Alqahtani, Fars K. Alanazi

**Affiliations:** 1 Department of Pharmaceutics, College of Pharmacy, King Saud University, Riyadh 11451, Saudi Arabia; ph.aalharbi@gmail.com (A.A.); msaalqahtani@ksu.edu.sa (M.S.A.); afars@ksu.edu.sa (F.K.A.); 2 Medicinal Aromatic and Poisonous Plants Research Center, College of Pharmacy, King Saud University, Riyadh 11451, Saudi Arabia; Fnasr@ksu.edu.sa (F.A.N.); onoman@KSU.EDU.SA (O.N.)

**Keywords:** bioactive self-nanoemulsifying drug delivery systems (Bio-SNEDDSs), solubility improvement, curcumin, thymoquinone, antioxidant activity, drug precipitation, MTT assay

## Abstract

Curcumin (CUR) is an attractive polyphenol for its anti-inflammatory, antibacterial, antioxidant, and anticancer properties. Poor solubility in water and sensitivity against sunlight are the most challenging characteristics in the development of CUR for clinical use. The aim is to develop oral lipid-based bioactive self-nanoemulsifying drug delivery systems (Bio-SNEDDSs) for curcumin as a candidate for cancer therapy. Bio-SNEDDSs containing black seed oil, medium-chain mono- and diglycerides, and surfactants were prepared as CUR delivery vehicles. The morphology, droplet size, physical stability, encapsulation efficiency, risk of precipitation, lipid digestion, antioxidant activity, and antimicrobial activity were evaluated for the representative formulations. Finally, an MTT assay was performed on MCF-7 cells to determine the cytotoxic effect of the different formulations. The results showed lower droplet size (28.53 nm) and higher drug-loading (CUR 20 mg, thymoquinone 1.2 mg) for the representative Bio-SNEDDS (black seed oil/Imwitor 988/KolliphorEL (35/15/50) % *w/w*), along with a transparent appearance upon aqueous dilution. The dynamic dispersion and in-vitro lipolysis data proved that the Bio-SNEDDS was able to keep the CUR in a solubilized form in the gastrointestinal tract. From the antioxidant and antimicrobial studies, it was suggested that the Bio-SNEDDS had the highest activity for disease control. The MTT assay showed that the representative Bio-SNEDDS treatment led to a reduction of cell viability of MCF-7 cells compared to pure CUR and conventional SNEDDSs. A Bio-SNEDDS with elevated entrapment efficiency, antioxidant/antimicrobial activities, and an antiproliferative effect could be the best anticancer drug candidate for potential oral delivery.

## 1. Introduction

Almost 90% of the drug candidates today suffer from low aqueous solubility and poor bioavailability. Due to these challenging issues, the formulation design for a particular drug has become more complex. To overcome these challenges, drug formulators must look to new formulation technologies that can ensure effective treatments for patients in need. Lipid-based self-nanoemulsifying drug delivery systems using bioactive oils (Bio-SNEDDSs) are attractive nanoparticle dosage forms that provide synergistic therapeutic effects in addition to increased solubility, and they enhance the intestinal absorption of poorly water-soluble drugs without decreasing their efficacy. Bio-SNEDDSs can be encapsulated in soft or hard gelatin capsules to maintain their physical and chemical stability and minimize plasma profile variations in patients.

The collection, identification, and evaluation of herbal medicine such as curcumin (CUR) as active pharmaceutical ingredients is challenging but also efficacious and authentic as herbal medicine has been used for centuries all over the world and has turned out to be the mainstay in the treatment of many diseases. In most cases, herbal medicine has less or no side effects compared to other conventional drugs and yields antioxidants and pharmacologically active compounds, which make herbal medicine more attractive to future treatment regimes [1].

**Figure 1:**
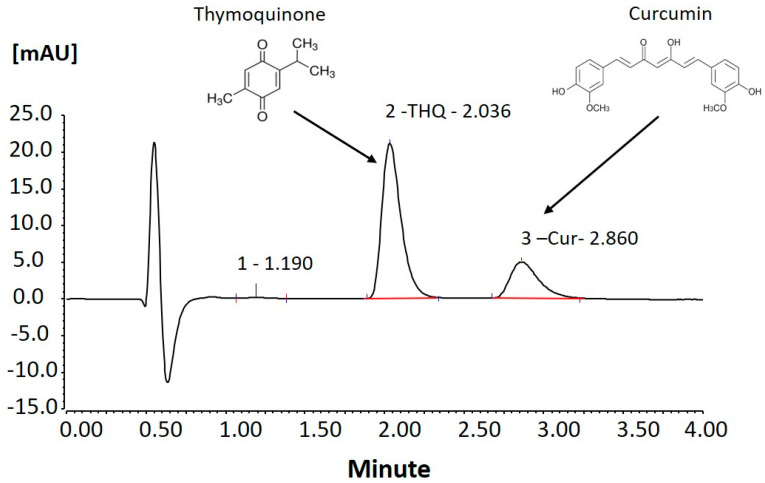
Chemical structure and UHPLC detection peaks of thymoquinone (MW-164.20, MP-41-51 °C) and curcumin (MW-368.38, MP-183 °C).

Curcumin (CUR; (1E,6E)-1,7-bis(4-hydroxy-3-methoxyphenyl)-1,6-heptadiene-3,5-dione) is a naturally occurring bright yellow pigment in turmeric, the ground rhizomes of *Curcuma longa* Linn (shown in Figure 1). CUR is the principal “curcuminoid” of turmeric, which has unique properties (such as a polyphenol) compared to other types of polyphenols such as “flavonoids” and “stilbenoids”. CUR has several pharmacological properties (recently proven), including antibacterial, anti-inflammatory, antioxidant, anticancer, antihyperlipidemic, and antidiabetic effects. It exerts potent anti-inflammatory effects, and these anti-inflammatory effects seem to be quite protective against some forms of cancer progression [2,3,4]. Several studies have been conducted that have proven the antimicrobial activity of CUR against some foodborne pathogens and spoilage microbes such as *Escherichia coli*, *Yersinia enterocolitica*, *Staphylococcus aureus*, *Bacillus subtilis*, *Bacillus cereus*, *Aspergillus niger*, *Penicillium notatum*, and *Saccharomyces cerevisiae* [5]. It exhibited a broad-spectrum inhibitory effect against all tested organisms by Oxford cup methods [6]. In addition, the antioxidant and anti-inflammatory effects of CUR could prevent Alzheimer’s disease as it significantly lowered oxidized proteins and interleukin-1ß, a proinflammatory cytokine that is elevated in the brains of APPsw transgenic mice (the model mice for Alzheimer’s disease) [7,8].

Black seed oil (BSO), which has been used as a bioactive excipient in formulation developments in current studies, is extracted from seeds of the herbaceous plant named *Nigella sativa.* BSO is widely used as indigenous traditional medicine for the treatment of arthritis, lung diseases, and hypercholesterolemia. Thymoquinone (THQ; 2-isopropyl-5-methylbenzo-1,4-quinone, shown in Figure 1) is the main phytochemical compound found in black seed oil, which has antioxidant effects and has been shown to protect against heart, liver, and kidney damage in animal studies as well as possibly having anticancer effects [9]. Some of the reported pharmacological properties of THQ include hypotensive, antinociceptive, uricosuric, choleretic, antifertility, antidiabetic, antihistaminic, antioxidant, anti-inflammatory, antimicrobial, antitumor, and immunomodulatory effects [10].

It also has analgesic and anticonvulsant effects in animal models and antiangiogenic and antiepileptic effects [11]. Furthermore, THQ could act as a free radical and superoxide radical scavenger and preserve the activity of various antioxidant enzymes such as catalase, glutathione peroxidase, and glutathione-S-transferase. The anticancer effects of THQ are mediated through different modes of action, including antiproliferation, apoptosis induction, cell cycle arrest, reactive oxygen species (ROS) generation, and antimetastasis/antiangiogenesis. The antitumor effects of THQ have also been investigated in tumor xenograft mice models for colon, prostate, pancreatic, and lung cancers [12,13,14].

The use of CUR and THQ as combination therapy could produce a greater therapeutic effect with the reduction of toxicity and other side effects related to high drug doses [15]. Coadministration of black seed and turmeric showed enhanced efficacy in preventing metabolic syndrome (MS) in fructose-fed rats. This study demonstrates the therapeutic superiority of the black seed and turmeric combination at low doses, over individually tested herbs, in improving features of MS [16]. Recently we used several bioactive components, including black seed oil, in the formulation design of a combined oral dosage form [9,17]. The studies were predominantly focused on drug loading and characterizations (in terms of appearance, droplet size, zeta potential) only, which was afterward converted into a solid dosage form to increase in-vitro drug release. However, the precise mechanism of synergistic action for the combined doses, particularly bioactive compounds, namely, THQ and CUR, are very poorly investigated. Therefore, to improve therapeutic selectivity, synergistic effects of drug combinations should be explored widely. Within the scope of the present investigations, the promising pharmacological properties of Bio-SNEDDSs could offer great therapeutic benefits to diseases such as cancer.

The bioactive self-nanoemulsifying drug delivery systems (Bio-SNEDDSs) in the current research were designed for CUR in a dosage form, with an expectation of complementary and synergistic therapeutic effects from black seed oil. In addition, the application was extended to antioxidant and cell viability studies to investigate the biological activity and cell death of MCF7.

## 2. Materials and Methods

### 2.1. Chemicals and Reagent

Curcumin (CUR, purity 99.9%) was purchased from Enzo Life Sciences (Lausen, Switzerland). THQ (purity > 99.8%) was obtained from Sigma Aldrich Co. (St Louis, MO, USA). Rutin hydrate (purity > 99.8%), 2,2-diphenyl-1-picrylhydrazyl (DPPH), and l-ascorbic acid were also purchased from Sigma Aldrich (St Louis, MO, USA). Imwitor 988 (I988, medium-chain mono-/diglycerides mix) and Kolliphor EL (KrEL) were purchased from BASF (Ludwigshafen, Germany). HCO-30 (PEG-30-hydrogenated castor oil) was a gift from Nikko Chemicals Co. (Tokyo, Japan). Black seed oil was extracted from naturally obtained *Nigella Sativa* seeds from Jessore, Bangladesh, by the cold-press method. MCF7 cancer cells were a kind gift from King Saud University’s cell culture lab (Riyadh, Saudi Arabia). Hanks balanced salt solution (HBSS), trypsin/EDTA solution, and RPMI 1640 medium and its components were obtained from Invitrogen (Waltham, MA, USA). HPLC-grade methanol (MeOH) and formic acid (FA) were obtained from BDH Chemicals Ltd. (Poole, UK). High purity Milli-Q water from a Milli-Q integral water purification system (Millipore, Bedford, MA, USA) was used throughout the experimental works. All other reagents were of analytical grade and used without further purification.

### 2.2. Methods

#### 2.2.1. Estimation of Thymoquinone Content (Phytochemical) in Black Seed Oil (BSO)

The extraction method of BSO was by the cold-press technique previously published by our group [18]. Approximately 500 g of seeds were collected from plants and sundried after cleaning with fresh water. Finally, the extracted oil was filtered and transferred to a screw-capped amber glass bottle until further use.

Thymoquinone (THQ) is the main bioactive constituent of BSO and, hence, was quantified using a standard analysis method. The 100 μg/mL stock solution of THQ was used as a reference standard for BSO standardization. The calibration curve, in the range of 0.1–50 μg/mL concentration of THQ, was constructed against absorbance. Briefly, 1 mL of BSO was separately dissolved in 10 mL of solvent in volumetric flasks to quantify the amount of THQ in BSO for our formulation. The amount of THQ in BSO was found to be approx. 3 mg/g, which was in accordance with those reported earlier in the literature [19].

#### 2.2.2. Gas Chromatography Mass Spectrometry (GC–MS) Analysis of BSO

GC–MS analysis was conducted using a Perkin Elmer model Clarus 600 T combined with a single quadrupole mass spectrometer (Perkin Elmer, MA, USA) and an Elite 5 MS column (30 m × 0.25 mm × 0.25 µm film thickness, (Perkin Elmer, MA, USA). The chromatographic analysis was performed at a flow rate of 1 mL/min, with high-purity helium as the gas carrier. The injector temperature was 280 °C and equipped with a split-less injector at 20:1; the sample injection volume was 1 microliter. The initial temperature was set to 40 °C and further increased to 150 and 300 °C at 10 °C min^−1^ (held for 1 min at every step). The MS ion source and inlet line temperature were set to 220 and 240 °C, respectively. The BSO was scanned at 40 to 600 mass ranges at 70 eV electron energy on a multiplier voltage of 270, with 4 min solvent delay. Finally, unknown constituents were identified by comparing the spectra with that of the Wiley 2006 library and NIST 2005 library (National Institute of Standard and Technology library). The total run time was 29 min for the analysis of a single sample.

#### 2.2.3. Curcumin and Thymoquinone Bio-SNEDDS Formulation Development

Bio-SNEDDS lipid-based formulations were developed using BSO and medium-chain mono-/diglycerides blends with two different nonionic surfactants, such as HCO30 and Kolliphor EL. The formulations were prepared with varying oil and surfactant concentrations by a simple preparation technique. Initially, a primary oil mixture was prepared with different oil varieties. Then, a surfactant was added to the oil at a fixed ratio. The final mixture was vortexed until homogeneity was achieved. The preconcentrate was kept in an airtight 3 mL glass tube until further use. Subsequently, the most interesting bioactive nanoemulsifying formulations (Bio-SNEDDSs) were considered for further investigation using model drug CUR. It is worth mentioning that bioactive material THQ was already present in the Bio-SNEDDSs at various concentrations, depending on the black seed oil concentration. Table 1 shows the composition of the formulations that were developed in the current study.

#### 2.2.4. Assessment of the Formulation Efficiency

The self-emulsifying efficiency of lipid-based formulations can be assessed simply by visual observation. It is a very common and time-saving technique used in the current study to prepare suitable SNEDDS formulations without making a great deal of trial and error [20,21]. Samples for visual observation were prepared using 100 mg of each anhydrous formulation dispersed into 100 mL of water in a beaker (1:1000 dilution). The diluted samples were agitated gently for 1 min at room temperature, and their miscibility, homogeneity, and appearance were assessed accordingly.

#### 2.2.5. Droplet Size and Polydispersity Index (PDI) Measurement

The droplet size of the nanoemulsifying systems is a factor that can facilitate faster drug release and rate of absorption due to their higher surface area in the gastrointestinal tract [22]. The droplet size and PDI of all the diluted formulations were measured by laser diffraction particle size analysis using a Malvern zetasizer (Model ZEN3600, Zetasizer Nano Series, Worcestershire, UK). The formulations were diluted at a ratio of 1:1000 *v*/*v* (formulation:distilled water, mixed for 1 min) and tested immediately. The diluted samples were placed directly into the cuvette and the data were collected 10 times. All the diluted samples, in triplicates, were placed into the cuvette and the data of each sample were collected 10 times.

#### 2.2.6. CUR and THQ Equilibrium Solubility

The equilibrium solubility of CUR + THQ in the lipid-based formulation was examined using a simple shake flask method. For sample preparation, an excess amount of drug was added to 1 g of each formulation, which was then vortexed for 3 min and occasionally agitated during the 7-day incubation period at 37 °C in a dry heat incubator. After 7 days, the samples were centrifuged at 13,000× *g* in 1.5-mL microfuge tubes to separate the undissolved drug from the formulations. An aliquot was weighed from the supernatant and diluted in methanol as solvent. The dissolved CUR and THQ were analyzed by using a previously developed UHPLC method by our group [19].

#### 2.2.7. Transmission Electron Microscopy (TEM)

The representative Bio-SNEDDS was investigated for its morphology using high-resolution TEM by Jeol (model JEM1010, Tokyo, Japan). Only the optimal nanodroplets/micellar systems containing no API were viewed using the microscope as there was a risk of crystallization of API, with subsequent damage to the microscope. Each nanodroplet/micellar solution was freshly prepared by diluting 10 times with distilled water, and a drop was placed on a carbon-coated copper grid. The Bio-SNEDDS component was stained with one drop of osmium and left to dry completely before loading into the microscope for viewing at 5000–20,000× magnification.

#### 2.2.8. Physical Stability Assessment of CUR Bio-SNEDDS

The two optimized SNEDDS/Bio-SNEDDS anhydrous formulations were assessed for their physical stability as a dosage form. The CUR was evaluated based on % of intact drug remaining in the formulation after 3 months of storage. In addition, the physical appearance of the anhydrous/diluted formulation was examined to record any turbidity, color change, or separation. The tested Bio-SNEDDS formulation was filled in airtight amber glass vials and stored at room temperature. A minimum of three replicates of each sample was analyzed at the initial period and 3 months, and the data were tabulated.

#### 2.2.9. Determination of Antimicrobial Activity

##### Disk Diffusion Method

The disk diffusion method was employed for the determination of the antimicrobial activity of the representative samples. Briefly, 100 μL of 10^7^ CFU/mL of bacterial suspension solutions in the exponential growth phase and 10^6^ CFU/mL of yeast were spread on Mueller–Hinton agar medium and Sabouraud dextrose agar, respectively [23]. Filter paper disks (9 mm of diameter) were impregnated with 20 μL of each sample (2 mg/disc) and placed on the inoculated Petri dishes. Negative control was performed using DMSO solvent, which was employed to dissolve the different samples. Ampicillin (2 mg/disc), kanamycin (2 mg/disc), and nystatin (2 mg/disc) were individually used as positive controls for bacteria and fungi. Petri dishes were then incubated for 24 h at 37 °C for bacterial strains and 48 h at 30 °C for fungi. Antimicrobial activity was evaluated by measuring the inhibition zone (mm) against the studied microorganisms, including disk diameter.

##### Estimation of Antioxidant Activity

Scavenging activity of DPPH radical: DPPH (2,2-diphenyl-1-picrylhydrazyl) was utilized to determine free radical scavenging by following the assay method described by Brand et al. (1995) [24]. This test was conducted to estimate the free radical scavenging ability of the Bio-SNEDDS formulation. Five different concentrations (10, 50, 100, 500, and 1000 μg/mL) of the representative samples were used in the experiment. Briefly, 1 mL of the test mixture was prepared using 500 μL of the formulation and 375 μL methanol, and 125 μL of 0.04% DPPH ethanolic solution was further added. Ascorbic acid was utilized as a positive control. After 30 min of incubation in the dark at room temperature, the reduction in absorbance was estimated at λ = 517 nm. The radical scavenging ability was determined from the equation:

% of radical scavenging activity = (Abs control − Abs sample/Abs control) × 100(1)

##### β-Carotene–Linoleic Acid Assay

The antioxidant activity of the samples was assessed by utilizing the *β*-carotene bleaching assay reported by Veligoglu et al. (1998) [25], with modifications. To flasks containing 0.02 mL of linoleic acid and 0.2 mL of Tween-20, 1 mL of a *β*-carotene solution (0.2 mg/mL in chloroform) was added. The chloroform was completely evaporated under vacuum pressure at 40 °C. The residue solution was immediately diluted with distilled water (100 mL) and swirled for 2 min to make an emulsion. A blank solution was prepared likewise but without the use of *β*-carotene. Additionally, a control (0.2 mL) was prepared containing 80% (*v*/*v*) methanol. Then, 5 mL of the emulsion was transferred into a tube containing 0.2 mL of the sample at 1 mg/mL. The sample tubes were incubated for 2 h in a water bath at 40 °C. Rutin (1 mg/mL) was used as a positive control. The absorbance of the sample was measured against the blank at 470 nm at 30 min intervals by UV–vis spectrophotometer (UV mini-1240, Shimadzu, Japan). The antioxidant activity was determined by utilizing the formula below:

% anti-oxidant activity = (Abs0 − Abst)**/**(Abs0 − Abst) × 100 (2)

where Abs0 and Abs0 are the absorbance values of the samples and control at zero time of incubation; Abst and Abst are the absorbance values of the samples and control after incubation for 120 min, respectively.

##### In Vitro Dynamic Dispersion Studies

Drug precipitation upon dispersion in the gastrointestinal tract (GIT) is a big limitation after a supersaturation system is achieved on solubility. If the free drug precipitates out of the aqueous solution, then the dose amount will be reduced, and the drug drops the driving force for systemic absorption. Therefore, assessing the risk of precipitation from supersaturated systems is highly required for a lipid-based self-nanoemulsifying formulation to keep the dosage form intact.

An amount of CUR was dissolved in the F4-Bio-SNEDDS and F7-SNEDDS at an 80% saturation level based on their equilibrium solubility data. Two optimized formulations from the equilibrium solubility studies were experimented on in the corresponding dynamic dispersion studies to investigate whether the drug would remain solubilized during aqueous dispersion in the GI tract. One gram of each formulation was dropped into 100 mL of fasted state simulated gastric fluid (FaSSGF) and fed state simulated intestinal fluid (FeSSIF). The dispersants were kept in an incubator at 37 °C for 24 h and 1 mL of the dispersed sample was withdrawn periodically (0 to 24 h) from each container. The withdrawn samples were then centrifuged at 13,000× *g*, and a 100-µL clear supernatant was assayed by the UHPLC method to estimate the percentage of drug that remained solubilized in the formulations. All of the experiments were performed in triplicate.

##### In Vitro Lipolysis Experiments

Lipolysis reaction tests were conducted for the optimized Bio-SNEDDS, with the pure drug as control. Briefly, 250 mg of the anhydrous formulation containing CUR and THQ (equivalent to 80% of equilibrium solubility) was dispersed into 9 mL of simulated intestinal aqueous media under fed conditions (FeSSIF). The FeSSIF was prepared using a lipolysis buffer containing phospholipid and bile salt at an actual secreted ratio in the intestine (20 and 5 mM). Prior to beginning the lipolysis reaction, the optimized Bio-SNEDDS was emulsified (stirred for 5 min) in a thermostatic-jacketed glass reaction vessel to form mixed micellar solutions [26].

Then, 1 mL pancreatic enzyme was prepared from the pancreatin containing 800 tributyrin units (TBU) of pancreatic lipase to start the reaction. The lipolysis reaction test was run for 30 min at 37 °C, using a pH-stat titration unit (Metrohm, Switzerland; adjusted pH 6.8). Samples were withdrawn periodically at 0 to 30 min during the titration reaction and directly analyzed by UHPLC systems to investigate the solubilized drug in digests.

##### Cell Viability (MTT Cytotoxic) Assay

MCF-7 cells (1 × 10^5^ cells; 1 mL) were plated in a 24-well plate and allowed to attach. After 24 h, cells were treated with the test concentrations (0–50 μg/mL) of each formulation in triplicate and incubated for 48 h. Thereafter, the media was removed and replaced with 100 μL MTT at 5 mg/mL. After 4 h incubation, 1 mL of acidified isopropanol was used to solubilize the MTT end product (formazan), and optical densities were measured with a plate reader (Bio-Tek; Elx-800, USA) at 570 nm. The average absorbance was used to determine the average cell viability, as follows:

% cell viability = Average absorbance of treated sample/Average absorbance of control × 100(3)

##### Statistical Analysis

The data were analyzed using Excel software (Excel 2013, Microsoft, Redmond, WA, USA). Data were expressed as mean ± standard error of mean (SEM). One-way ANOVA was applied to determine the significance of the value. *P*-values < 0.05 were considered significant throughout the studies.

## 3. Results and Discussion

### 3.1. Chemical Constituents of BSO

**Figure 2:**
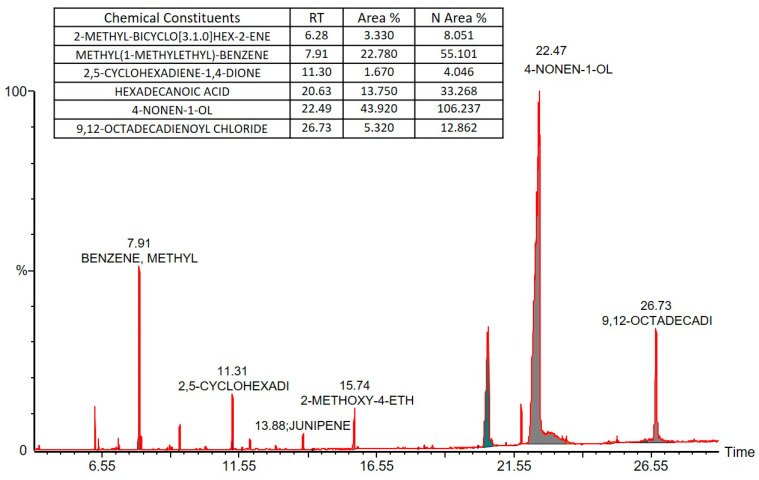
GC–MS chromatogram of black seed oil extract and the list of the major chemical constituents.

The BSO, which was extracted from *Nigella sativa*, was further analyzed by GC–MS chromatography to identify the chemical constituents of the cold-pressed oil that were soluble in organic solvent methanol (Figure 2). The chemicals that were found in substantial quantities are listed in an inset table. 4-Nonen 1-ol was found in the high concentrations, along with methyl (1-methylethyl)-benzene and hexadecanoic acid (a common saturated fatty acid), and our work then focused on evaluating the activity of the extracted oil using bioactive self-nanoemulsifying drug delivery systems (Bio-SNEDDSs) in vitro.

### 3.2. Visual Assessment, Dispersion Time, Droplet Size, and PDI Analysis

Visual observation and droplet size analysis results of the lipid-based formulations (F1 and F7) in the current study are shown in Table 1. F1, the oil-only formulation, and F2, BSO with Imwitor 988 (mono- and diglyceride mix) produced a turbid appearance upon dilution with water. The F1 formulation was not miscible with water and, therefore, was tagged as nondispersed. These formulations produced larger particle sizes of around 2 µm upon aqueous dispersion and are not categorized as SNEDDSs. On the other hand, F3, F5, and F6 formulations, which were blended with surfactant HCO-30 (HLB 11.5), produced a bluish appearance and dispersed within a minute. These three formulations produced the nano-ranged particle size of 75.22, 81.09, and 102.41 nm, with moderate values of polydispersity index, respectively. In addition, F4 and F7 were the best formulations in terms of their transparent appearances and smaller droplet sizes. These two formulations produced particle sizes of less than 30 nm, with very low polydispersity index values. The formulations F3–F7 are categorized as nanoemulsifying formulations and were further experimented on in various other studies. The light-yellow color of the SNEDDS became intense with an increase in the amount of black seed oil in the formulations of F1 to F6 due to the black color of BSO.

SNEDDSs are able to readily form an emulsion upon gentle agitation. Due to the presence of surfactants, the formation of an emulsion is considered spontaneous when the Gibbs free energy is at a low positive or even negative value. Surfactants surround the droplet’s oily phase and reduce the interfacial energy. It was suggested that the droplet formation can emerge by the mere erosion of a small droplet from a larger one [27].

The objective of conducting the visual assessment tests was to optimize the formulation that could provide good miscibility and homogeneity, faster dispersion time, and bluish-transparent type appearance upon 1 in 1000 dilution with water. Visual observation can be the prime initial indicator for distinguishing good and bad formulations and is sufficient for an experienced formulator to reduce trial and error. Within the scope of the current study, if the formulations were homogeneous and took considerably less than a min to disperse, they were considered to be efficient. The results of the visual assessment showed that F3–F7 formulations were found to be effective in terms of their appearances, droplet sizes/PDI, and emulsification times.

Agreeing with previous studies, the self-emulsifying efficiency of a lipid-based formulation is strongly associated with the average droplet size upon aqueous dispersion [28,29]. However, for lipid-based oral drug delivery, there are two main factors that commonly describe the efficient systems: (a) the rate of emulsification and (b) the particle size distribution of the subsequent emulsion.

Self-emulsification ability becomes the main point in SNEDDSs and/or Bio-SNEDDS evaluation because bioavailability and the oral absorption efficiency of a practically insoluble drug can be increased by a self-emulsification process that generates fine dispersion and micellar solution to avoid drug precipitation and recrystallization [30]. The observation data indicated that formulations F2–F6 have a good self-nanoemulsification ability and completely dispersed in less than a minute when diluted with water, with a little help from low agitation of peristaltic activity [31].

### 3.3. Effect of Surfactant (HCO30) on Particle Size

**Figure 3:**
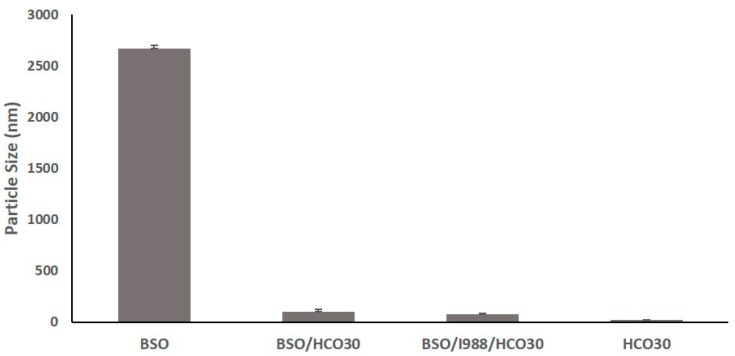
Effect of surfactant on the droplet size of the curcumin (CUR) bioactive formulations. The excipients were mixed as % (*w*/*w*). The formulations were represented by BSO (100%)-F1, BSO/HCO30 (50/50%)-F6, BSO/I988/HCO30 (35/15/50)-F3, and HCO30-F7, respectively.

Nonionic surfactants and their compatibility with lipid-based excipients play a significant role in reducing the particle size of the self-nanoemulsifying formulations. The data from the droplet size analysis of oil and surfactant mixtures showed that if a surfactant is the only excipient added in the formulations, the particle size reduces dramatically (Figure 3). In addition, the increased amount of surfactant concentration in the formulations could also further reduce the droplet sizes. These data were proven in accordance with the previously published results of our group [17].

### 3.4. Effect of Surfactant on the Solubility of CUR

**Figure 4:**
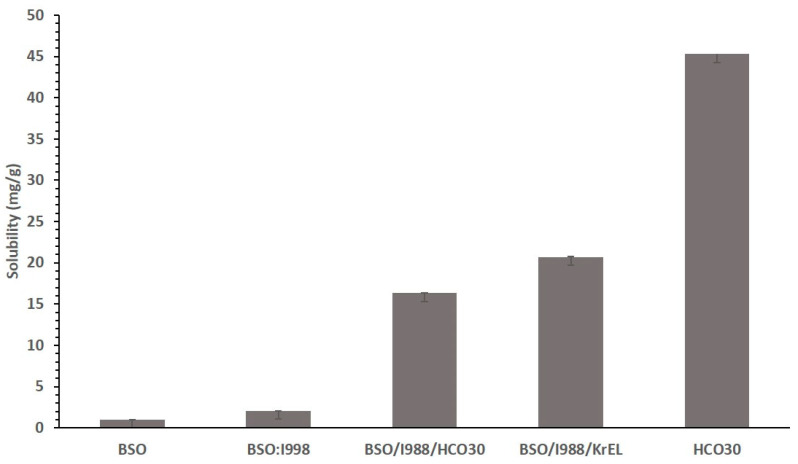
Effect of surfactants on the solubility of the CUR bioactive formulations. The excipients were mixed as % (*w*/*w*). The formulations were represented by BSO (100%)-F1, BSO/I988 (70/30%)-F2, BSO/I988/HCO30 (35/15/50%)-F3, BSO/I988/KrEL (35/15/50%)-F4, and HCO30 (100%)-F7, respectively.

The nature of the nonionic surfactant is an important factor affecting the solubility of Bio-SNEDDSs. The results in Figure 4 show that the surfactant-only formulation (F7) solubilized the highest amount of CUR compared to all other formulations. The lowest drug loading was observed in the case of oil-only formulation, BSO. The higher content of surfactant in the formulations exhibited increased CUR solubility. The overall results from Figure 3 confirmed that CUR is more suitable for surfactant reach formulations. However, formulation scientists should be aware of the usage of a higher percent of surfactant, which may cause irritation in the stomach.

### 3.5. Equilibrium Solubility Studies

The equilibrium solubility experiments were carried out to estimate the maximum dose level of CUR in a single unit capsule/tablet. Thymoquinone (THQ), a property of BSO, was naturally present in the BSO-containing formulations; hence, it was also estimated along with CUR. All the seven anhydrous formulations were kept in an incubator for a seven-day period, with occasional agitation, if needed, to confirm that the equilibrium had been achieved. Equilibrium solubility/drug loading of CUR and THQ in various formulations is presented in Table 2.

Among all the formulations, CUR was significantly soluble in F7 systems (solubility was 45.30 mg/g). The lowest solubility of CUR was found in formulations F1, and F2, which were reached in BSO. However, these two formulations had a comparably high quantity of THQ. On the other hand, F4 systems solubilized 20.695 mg/g CUR with 1.207 mg/g THQ in the formulation. Similarly, F3, F5, and F6 formulations were able to solubilize 14 to 16 mg/g CUR and 0.8–1.2 mg/g THQ. The higher solubility in formulations F4 and F7 suggests that CUR can be dissolved in a higher amount in water-soluble materials.

From the overall solubility data, it was confirmed that higher drug solubility and better aqueous dispersibility were reported for the F4-Bio-SNEDDS (transparent; BSO:I988 (7:3)/KolliphorEL (1/1 % *w*/*w*)) and the F7-SNEDDS (surfactant-only formulation; HCO-30) and chosen for further experimental cell line studies, in vitro dispersion, and digestion studies for CUR. The study also established that CUR is preferably dissolved at higher amounts in the formulations containing more polar mixed glycerides and water-soluble nonionic surfactants.

### 3.6. Physical Stability Assessment of CUR Bio-SNEDDS

**Figure 5:**
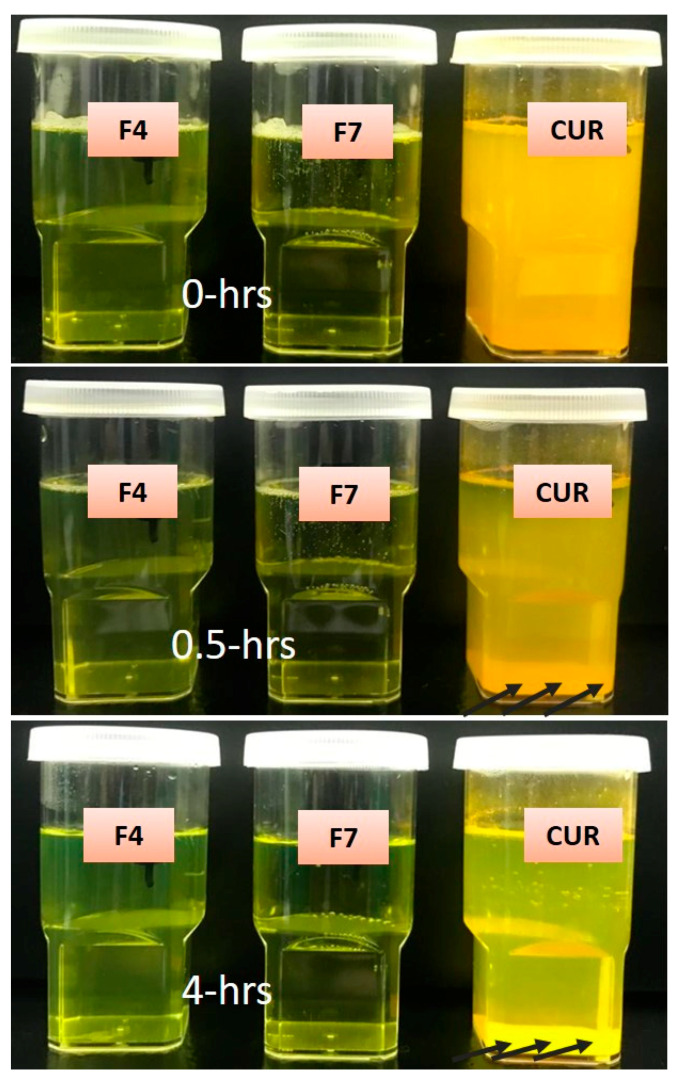
Aqueous dispersion shows the physical stability of formulation F4 (BSO/I988/KolliphorEL (35/15/50 % *w*/*w*)), F7 (HCO-30), and pure CUR powder at 0, 0.5, and 4 h periods at room temperature. The arrow shows the precipitation of CUR powder.

A physical stability assessment was carried out to determine the standard storage period where the Bio-SNEDDS remains intact without making any phase separation at aqueous dilution (creaming or cracking) phases. The test results showed that the anhydrous CUR Bio-SNEDDS formulation (F4) did not show any phase separation, viscosity change, or change in appearance upon storage for 3 months. In addition, the surfactant-only SNEDDS formulation (F7) also did not change its appearance/viscosity from the initial dispersion. In addition, an aqueous dispersion of anhydrous F4 and F7 formulations in Figure 5 showed no drug precipitations and appearance changes. In comparison, pure CUR powder was significantly precipitated when diluted with water, almost immediately (within 30 min). This proves the statement of the previous studies conducted by Hintzen F. et al. [32] that the SNEDDS dosage form has good physical stability for a substantial period of time in both anhydrous and diluted forms. This was due to the use of a nonionic surfactant (hydrogenated castor oil) in the Bio-SNEDDS, which dissolved hydrophilic surfaces and drugs in the oil phase and, thus, improved the stability by forming bilayer rigidity of the system [33].

The optimized Bio-SNEDDS (F4) and SNEDDS (surfactant-only formulation F7), along with pure CUR powder, were tested to examine the effect of time upon storage for a three-month period. Table 3 presents the appearance, particle size, zeta potential value, and drug amount of the formulation at 0- and 3-months’ time. The data in Table 3 show a small decrease in percent drug without any changes in appearance from the initial to three-month period in both F4 and F7 formulations. However, the particle size was slightly decreased in F7, which could be due to the minor precipitation of CUR from the SNEDDS. The zeta potential (ZP) values of F4 was almost unchanged, which confirmed that the formulation was stable enough for three months. However, the ZP value of F7 was not consistent and increased in the three-month period. The overall results show the better performance of F4 Bio-SNEDDS based on the retention of drug content and characteristic features, although the data for both formulations were within the acceptance criteria.

### 3.7. Transmission Electron Microscopy (TEM)

**Figure 6:**
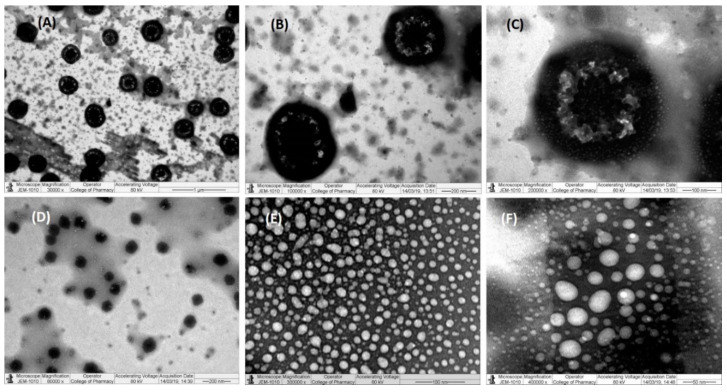
TEM micrographs of CUR-loaded Bio-SNEDDS/SNEDDS. (**A**–**F**) Images at different magnifications of F4 Bio-SNEDDS and F7 SNEDDS, respectively.

As shown in the transmission electron microscopy (TEM) images (Figure 6), the nanoemulsions of F4 and F7 exhibited a spherical shape structure with varying sizes. The shape and size of the droplets were evident from the particle size data obtained from Malvern zetasizer measurements of less than 30 nm. Such findings prove the spontaneous formation of nanometer-ranged nanoemulsifying systems.

TEM is a determining technique for studying microstructures due to the production of high-resolution images, and it can capture any transitions of the structure. Only the optimal CUR-loaded nanoemulsifying systems (F4 Bio-SNEDDS and F7 SNEDDS; free from precipitations) were analyzed as there was a risk of crystallization of CUR with other formulations, which could subsequently damage the microscope.

### 3.8. In Vitro Dynamic Dispersion Studies

**Figure 7:**
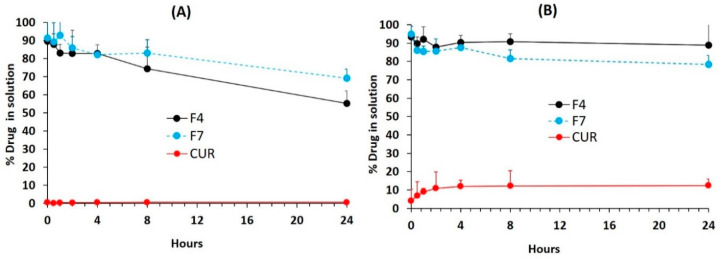
Percentage of the original dose of CUR remaining in solution after 1:100 dilutions of the formulations F4 Bio-SNEDDS (BSO/I988/KolliphorEL (35/15/50 % *w*/*w*)), F7 SNEDDS (HCO-30), and CUR pure powder in (**A**) FaSSGF and (**B**) FeSSIF dispersion medium (the CUR concentration in the dose was 80% of equilibrium solubility in the anhydrous formulation). Data are presented as mean ± SD (*n* = 3).

In order to investigate how long the concentration of curcumin (CUR) remains in the solution or whether quick precipitation is likely to occur for the representative F4-Bio-SNEDDS formulation and its counterpart F7-SNEDDS formulation, in vitro dispersion tests were carried out and compared with pure CUR powder as control. Both F4 and F7 were loaded with 16.5 and 36.2 mg/g CUR, an amount equivalent to 80% of equilibrium solubility. Figure 7 shows the % CUR remaining in solution at 0, 0.5, 1, 2, 4, 8, and 24 h after the dispersion of the formulation in fasted state simulated gastric fluid (FaSSGF) and fed state simulated intestinal fluid (FeSSIF at pH 5.0) at 37 °C. The test also indicates the concentration of CUR that was in a supersaturated state immediately after the dilution takes place. The data from the FaSSGF in Figure 7A showed that F4 Bio-SNEDDS (the blend of BSO and I988 with Kolliphor EL) was able to maintain approx. 80% CUR in solution up to 4 h and continuously precipitated up to 24 h. On the other hand, in FeSSIF media (Figure 7B), F4 Bio-SNEDDS kept more than 90% CUR in solution up to 4 h without making further precipitations up to 24 h. The data from FeSSIF media suggest that CUR was supersaturated in the presence of the bile salt/phospholipid solution secreted in the small intestine, which was absent in the stomach. However, F7, as a surfactant-rich formulation, was unable to hold CUR in the same manner. In comparison, the pure CUR was precipitated almost immediately, although 10% remained solubilized in FeSSIF media.

The dynamic dispersion test is a major in-vitro tool to predict the fate of the drug during transit through the gastrointestinal (GI) tract, up to the systemic circulation. However, formulation scientists should consider using simulated intestinal aqueous media to conduct the dispersion test for better correlation. Previous studies suggest that if the poorly soluble drug stays in solution by maintaining a supersaturation level until its absorption, it could have a better performance in vivo [34,35,36]. SNEDDSs that disperse within few seconds to produce a transparent appearance with reduced droplet sizes might fail and or lose its advantage if the drug precipitates out of the solutions in the GI tract. In this study, we have focused on the dispersion phase in stomach (FaSSGF) and intestinal media (FeSSIF) and investigated the influence of formulation excipients on drug precipitation.

Previous studies have confirmed that the absorption of the drug from the supersaturation state was consistent in humans under gastric and intestinal conditions and, therefore, indicate the potential significance of this approach, which could be predicted from an in-vitro precipitation study. However, if supersaturation affects the absorption of poorly soluble drugs through first-pass metabolism, a combination with an in-vivo study should be required to assess its impact on oral bioavailability [37,38].

### 3.9. In Vitro Lipolysis Studies

From past experimental studies, we strongly believe that in vitro lipolysis testing should be used in parallel with dispersion/precipitation testing to predict the in-vivo performance of SNEDDS formulations.

**Figure 8:**
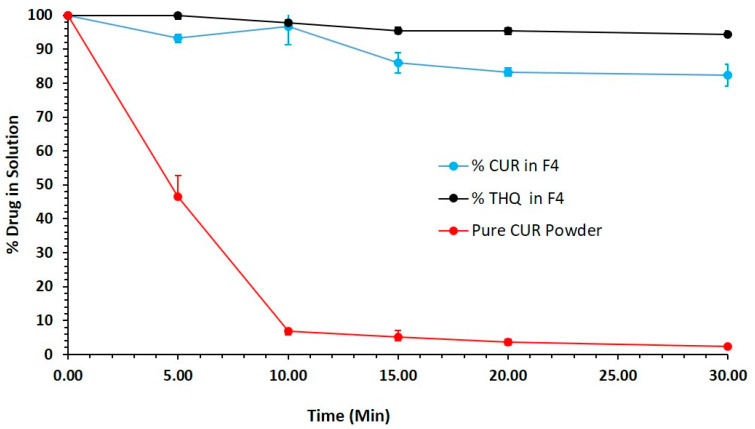
Percentage of the drug dose in solution in the presence of pancreatic lipase from the representative Bio-SNEDDS formulation (F4-BSO/I988/KolliphorEL (35/15/50 % *w/w*)), which contains CUR and THQ in a combined dosage form in FeSSIF solution during the in-vitro digestion experiment at various time intervals within a 30-min reaction period.

Figure 8 shows the 30 min lipolysis reaction of the Bio-SNEDDS formulation and pure CUR powder. The data illustrates that the representative Bio-SNEDDS (BSO/I988/KolliphorEL (35/15/50 % *w*/*w*)) formulation maintained more than 90% CUR in solution during the intestinal lipid–enzymatic reaction (lipid digestion). In addition, THQ was solubilized by almost 99% in the lipid digests of the Bio-SNEDDS. Alternatively, pure CUR showed dramatic precipitation almost immediately after the reaction started. The dynamic in-vitro techniques for lipid dispersion and digestion, when used simultaneously, offer the formulator the great advantage of predicting the likelihood of drug precipitation in both the stomach and intestine. The extent of in vitro–in vivo correlation (IVIVC) needs to be studied widely to generate more experiential data of lipid-based delivery systems, which will be valuable tools for formulators [39].

### 3.10. Antimicrobial Activity

#### 3.10.1. Disk Diffusion Assays

**Figure 9:**
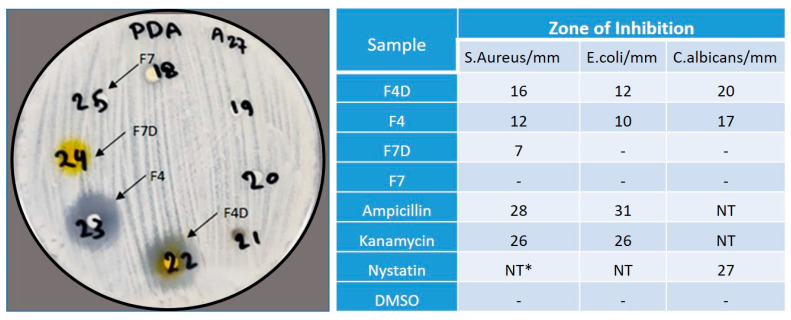
Antimicrobial activities of the representative F4-(BSO/I988/KolliphorEL (35/15/50 *%w/w*)) and F7-(HCO-30), with and without CUR, against *Staph. aureus*, *E. coli,* and *C. albicans.* F4 and F7 denote the blank formulations, and F4D and F7D the drug-loaded formulations, respectively. NT*, not tested.

F4D (drug-loaded) showed moderate activity against both Gram-positive and Gram-negative bacteria and strong antifungal activity against *C. albicans* (Figure 9). It was also observed that F4 (drug-free Bio-SNEDDS) showed excellent antibacterial and antifungal activities, indicating that thymoquinone (THQ) possesses strong antibacterial and antifungal properties. On the other hand, F7D (drug-loaded) showed antimicrobial activity against Gram-positive bacteria, while F7 (drug-free) showed no activity, indicating that curcumin was responsible for the antimicrobial activity in F7D. No inhibition was observed with DMSO solvent, which was used as a control. Bacterial and fungal growth was inhibited by antibiotics and used as control. Ampicillin inhibition zones varied from 28 mm for *Staph. aureus* to 31 mm for *E. coli*; Kanamycin inhibition zones ranged between 26 mm for *Staph. aureus* and 26 mm for *E. coli*, and the Nystatin inhibition zone was 27 mm for *C. albicans*.

#### 3.10.2. Antioxidant Activity

The results of the radical scavenging and antioxidant activities are shown in Table 4. In the *β*-carotene-bleaching model system, the samples showed variable powers of inhibiting *β*-carotene bleaching at a concentration of 1000 μg/mL, with total antioxidative values of 57.2, 48.3, 19.3, and 2.6 for F4D, F7D, F4, and F7, respectively (Table 4). In addition to that, the results of the DPPH radical scavenging method demonstrated comparable free-radical scavenging activity for the four formulation samples. Moreover, the CUR-loaded Bio-SNEDDS (F4D) exhibited the highest antioxidant and free-radical scavenging activity among the tested samples, with a value of 68.7 at 1000 μg/mL.

#### 3.10.3. Bio-SNEDDS Enhances Curcumin-Induced Inhibition of MCF-7 Cell Growth

**Figure 10:**
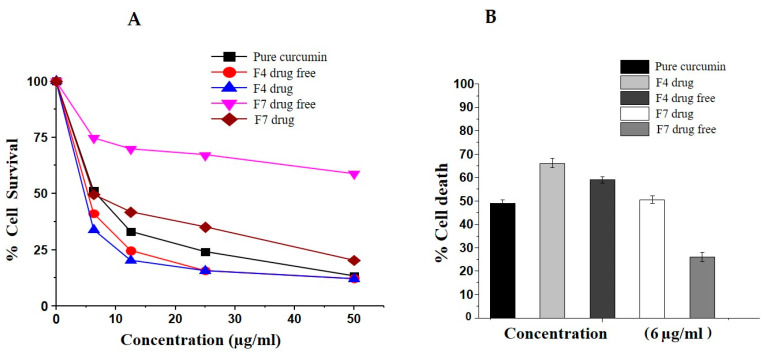
Effect of formulations on MCF-7 cell viability determined by MTT assay. (**A**) MCF-7 cells treated with various concentrations (0, 6, 12.5, 25, and 50 μg/mL) for 48 h. (**B**) The percentage of cell death after treatment with 6 μg/mL of each formulation. Data are expressed as the mean ± SD of three independent experiments.

The inhibitory effect of all the drug-free and drug-loaded formulations and the control (pure CUR powder) on the growth of MCF-7 cells was examined by MTT assay. The cytotoxic effect in terms of IC_50_ (the concentration that kills 50% of cells) was calculated from the dose-dependent curve using OriginPro 8.5 software (Table 5). As shown in Figure 9, all the formulations induced cytotoxicity against MCF-7 cells in a concentration-dependent manner; however, F4-Bio-SNEDDS (drug-loaded) exerted the greatest inhibitory effect in comparison to all other formulations. Additionally, MCF-7 cell treatments with all formulations at the same concentration (6 μg/mL) also showed that drug-loaded F4-Bio-SNEDDS had the most activity, followed by F4-Bio-SNEDDS (drug-free), F7-SNEDDS (drug-loaded), pure CUR, and F7-SNEDDS (drug-free), respectively (Figure 10B).

The overall results from the studies suggest that F4 Bio-SNEDDS provided synergistic effects due to the presence of thymoquinone, which could play a significant role in the % cell death. The current findings are in agreement with previously reported data that have suggested that curcumin inhibits MCF-7 cell proliferation, either alone or in combination with anticancer drugs [40]. Increasing evidence indicates that curcumin (CUR) has potential therapeutic activities against breast cancer MCF7 cells through multiple signaling pathways [41]. To date, no studies have explored its potential antiproliferation effects with thymoquinone. Therefore, in the current study, we tried to investigate the effect of curcumin in combination with thymoquinone on MCF-7 cell viability.

## 4. Conclusions

Bio-SNEDDSs were successfully developed using black seed oil as a well-recognized bioactive component for the oral delivery of curcumin. The representative Bio-SNEDDS (BSO/I988/KolliphorEL (35/15/50 % *w*/*w*)) produced stable uniformed nanodroplets with a transparent appearance and proved to be an effective carrier by keeping the curcumin (≥90%) in a solubilized form during dispersion and digestion within the GI tract. Our findings also demonstrate that curcumin with thymoquinone has better antimicrobial and antioxidant activities when formulated as a Bio-SNEDDS delivery system. Accordingly, the enhancement of the antiproliferative effect of the curcumin-loaded Bio-SNEDDS, evident in the present work, suggests its potential synergistic combination in cancer therapy.

## Figures and Tables

**Table 1:** The effect of compositions on the appearance of various SNEDDS formulations prepared for CUR oral delivery.

No	Formulation Compositions	Appearance	Spontaneity	Particle Size (nm)	PDI
F1	BSO	Turbid	nondispersed	2668.50 ± 32.11	1.00
F2	BSO:I998 (7:3)	Turbid	<1 min	1934.54 ± 15.02	1.00
F3	BSO:I988 (7:3)/HCO30 (1/1)	Bluish	<1 min	75.22 ± 8.34	0.694
F4	BSO:I988 (7:3)/KolliphorEL (1/1)	Transparent	<1 min	28.53 ± 0.18	0.129
F5	BSO:I988 (1:1)/HCO30 (1/1)	Bluish	<1 min	81.09 ± 15.12	0.447
F6	BSO/HCO30 (1/1)	Bluish	<1 min	102.41 ± 17.89	0.651
F7	HCO30	Transparent	1–5 min	19.81 ± 0.39	0.186

**Table 2:** Equilibrium solubility of curcumin (CUR) and thymoquinone (THQ estimated from BSO) in various SNEDDS/Bio-SNEDDS formulations (*n* = 3, mean ± SD).

No.	Formulation Ratios (% *w*/*w*)	Solubility of CUR (mg/g)	Solubility of THQ (mg/g)
F1	BSO	0.977 ± 0.013	3.014 ± 0.018
F2	BSO:I998 (7:3)	2.066 ± 0.006	2.094 ± 0.010
F3	BSO:I988 (7:3)/HCO30 (1/1)	16.328 ± 0.049	1.266 ± 0.005
F4	BSO:I988 (7:3)/KolliphorEL (1/1)	20.695 ± 0.052	1.207 ± 0.005
F5	BSO:I988 (1:1)/HCO30 (1/1)	15.276 ± 0.064	0.873 ± 0.005
F6	BSO/HCO30 (1/1)	14.554 ± 0.036	1.273 ± 0.011
F7	HCO30	45.300± 0.049	NP

Abbreviations: BSO, black seed oil; HCO-30, PEG-30 hydrogenated castor oil; I988, Imwitor 988; Kolliphor EL, hydrogenated castor oil (polyoxyethelene-35). NP: not present.

**Table 3:** Time-dependent stability studies showing the droplet size (upon dispersion) and drug recovery after three months at room temperature. CUR dissolved in the formulations is equivalent to 80% of equilibrium solubility.

Formulation	0 Months			3 Months		
	Drug %	Z-Ave (d.nm)	ZP. (mV)	Drug %	Z-Ave (d.nm)	ZP. (mV)
F4	100%	28.53 ± 0.18	−22.17 ± 2.90	97.36 ± 3.45	28.31 ± 0.87	−21.59 ± 1.89
F7	100%	19.81 ± 0.39	−10.39 ± 0.66	93.88 ± 1.62	18.99 ± 0.43	−12.32 ± 1.22
	Appearance					
F4	Transparent			Transparent		
F7	Transparent			Transparent		

**Table 4:** 2,2-diphenyl-1-picrylhydrazyl (DPPH) scavenging and antioxidant activities of F4-(BSO/I988/KolliphorEL (35/15/50 % *w/w*)) and F7-(HCO-30), with and without CUR. F4 and F7 denote the blank formulations, and F4D and F7D the drug-loaded formulations, respectively.

Sample	(%) Antioxidant Activity against Conc. (1000 µg/mL)	Radical Scavenging Activity (%DPPH against Conc.)				
		10 (µg/mL)	50 (µg/mL)	100 (µg/mL)	500 (µg/mL)	1000 (µg/mL)
F4D	57.2 ± 2.8	12.7 ± 4.1	20.3 ± 4.3	34.3 ± 3.9	56.3 ± 4.1	68.7 ± 3.2
F4	19.3 ± 1.4	-	-	-	-	21.3 ± 2.4
F7D	48.3 ± 3.2	6.21 ± 4.2	12.2 ± 3.1	22.3 ± 2.4	38.3 ± 4.6	51.6 ± 3.7
F7	2.6 ± 2.8	-	-	-	-	3.7 ± 2.2
Ascorbic acid	NT	80.7 ± 2.5	85.1 ± 1.3	85 ± 3.2	88.7 ± 2.7	90.7 ± 4.4
Rutin	89.3	NT	NT	NT	NT	NT

β-carotene bleaching assay, NT, not tested. Data are expressed as means ± SD and are significant at *p* < 0.05 (*n* = 3).

**Table 5:** The effect of the MTT assay on the formulations to evaluate IC_50_ (the concentration that kills 50% of cells).

Sample	MCF-7 IC_50_ (µg/mL)
Pure CUR control	6.67 ± 0.5
Drug-loaded F4 Bio-SNEDDS	4.76 ± 0.3
Drug-free F4 Bio-SNEDDS	5.2 ± 0.4
Drug-loaded F7 SNEDDS	5.94 ± 0.5
Drug-free F7 SNEDDS	>50
